# Clinical Outcomes in Routine Evaluation Measures Following Utilization of Peer Support and Supportive Text Messaging in Mental Health- Controlled Observational Study

**DOI:** 10.1192/j.eurpsy.2023.241

**Published:** 2023-07-19

**Authors:** R. Shalaby, V. Agyapong

**Affiliations:** Department of Psychiatry, University of Alberta, Edmonton, Canada

## Abstract

**Introduction:**

Peer support workers (PSW) and text messaging services (TxM) are effective mental health supportive services. Both interventions have positive outcomes, with TxM demonstrating clinical and economic effectiveness and PSW showing its utility within the recovery-oriented model.

**Objectives:**

To evaluate the effectiveness of combining PSW and TxM together in reducing psychological distress of recently discharged patients receiving psychiatric care.

**Methods:**

In a prospective, rater-blinded, pilot-controlled observational design, 181 discharged patients were recruited and randomized into four conditions; TxM only, PSW only, TxM and PSW, and treatment as usual. Clinical Outcomes in Routine Evaluation-Outcome Measure (CORE-OM), a standardized measure of mental distress, was examined at four time points: baseline, six weeks, three months, and six months. MANCOVA was used to assess the impact of the interventions on participants’ scores on four CORE-OM subscales across the three follow-up time points.

**Results:**

A total of 63 patients completed assessments at each time point. The interaction between PSW and TxM was predictive of differences in scores on the CORE-OM functioning subscale with a medium effect size (F1,63 = 4.19; p = 0.045; ηp2 = 0.07). The PSW + TxM group consistently achieved higher rates of recovery and clinical and reliable improvement compared to the other study groups. Additionally, the text message group and the PSW + TxM group significantly reduced the prevalence of risk of self/other harm symptoms after six months of intervention, with 27.59% (χ2(1) = 4.42, p = 0.04) and 50% (χ2(1) = 9.03, p &lt; 0.01) prevalence reduction, respectively.

**Conclusions:**

Combining PSW and TxM is effective with positive clinical outcomes for acute care patients. Incorporating the two interventions into routine psychiatric care for patients after discharge is highly recommended.

**Disclosure of Interest:**

None Declared

